# Functional near-infrared spectroscopy-based diagnosis support system for distinguishing between mild and severe depression using machine learning approaches

**DOI:** 10.1117/1.NPh.11.2.025001

**Published:** 2024-04-24

**Authors:** Zhiyong Huang, Man Liu, Hui Yang, Mengyao Wang, Yunlan Zhao, Xiao Han, Huan Chen, Yaju Feng

**Affiliations:** a Chongqing University, School of Microelectronics and Communication Engineering, Chongqing, China; b Chongqing University Central Hospital, Chongqing Emergency Medical Center, Chongqing, China; c Chongqing Mental Health Center, Department of Clinical Psychology, Chongqing, China

**Keywords:** severity of depression, functional near-infrared spectroscopy, machine learning, CEEMDAN-WPT

## Abstract

**Significance:**

Early diagnosis of depression is crucial for effective treatment. Our study utilizes functional near-infrared spectroscopy (fNIRS) and machine learning to accurately classify mild and severe depression, providing an objective auxiliary diagnostic tool for mental health workers.

**Aim:**

Develop prediction models to distinguish between severe and mild depression using fNIRS data.

**Approach:**

We collected the fNIRS data from 140 subjects and applied a complete ensemble empirical mode decomposition with an adaptive noise-wavelet threshold combined denoising method (CEEMDAN-WPT) to remove noise during the verbal fluency task. The temporal features (TF) and correlation features (CF) from 18 prefrontal lobe channels of subjects were extracted as predictors. Using recursive feature elimination with cross-validation, we identified optimal TF or CF and examined their role in distinguishing between severe and mild depression. Machine learning algorithms were used for classification.

**Results:**

The combination of TF and CF as inputs for the prediction model yielded higher classification accuracy than using either TF or CF alone. Among the prediction models, the SVM-based model demonstrates excellent performance in nested cross-validation, achieving an accuracy rate of 92.8%.

**Conclusions:**

The proposed model can effectively distinguish mild depression from severe depression.

## 1 Introduction

Depression is a prevalent psychological disorder characterized by persistent feelings of sadness and loss of interest in activities, significantly impacting the lives and work of individuals during the onset of the illness. Recognized as a pressing concern by the WHO’s Mental Health Gap Action Program (mhGAP),^1^ ∼3.8% of the global population suffers from this condition. The severity of depression, as assessed by the Hamilton Depression Rating Scale (HAMD),^2^ can be roughly categorized as mild, moderate, and severe. However, the diagnosis process often involves subjective evaluations, such as clinical interviews and professional medical assessments, which may impede the timely and accurate diagnosis of severe depression. Moreover, due to the shortage of mental health workers, only 9.2% of individuals with depression receive appropriate treatment, whereas some individuals with anxiety disorders or mild depression are mistakenly prescribed antidepressant medications.^3^ Research^4^ has indicated that antidepressants are ineffective in treating mild depression. Thus, there is an urgent need to develop an objective and efficient approach to quickly differentiate between mild and major depression for early diagnosis and effective treatment evaluation.

Several studies^5^^,^^6^ have found evidence of structural and functional changes in various brain regions among individuals with severe depression. These changes include abnormal activity in prefrontal, limbic, thalamic, and cortical areas. In prefrontal regions, increased depression severity is associated with decreased neural activity in the low-frequency range and increased activity in the high-frequency range.^7^ In addition, severe depression is characterized by extremely low mood, which may be accompanied by severe sleep problems, loss of appetite, and loss of interest in daily activities. Even for mild depression, the changes in the brain and physiology may be relatively mild but can still lead to problems with mood, concentration, and cognition.^8^^,^^9^ Depression severity has been found to be significantly associated with cognitive performance in situational memory, executive functioning, and processing speed, and as depression severity increases, these cognitive abilities decline.^10^ Impaired cognitive function in depressed patients results in reduced regional cerebral blood flow (rCBF) in the frontal, temporal, and anterior cingulate gyrus during cognitive tasks.^11^ Functional near-infrared spectroscopy (fNIRS) serves as a non-invasive, portable tool for monitoring hemodynamic responses in the cerebral cortex, commonly utilized to observe hemodynamic changes in psychiatric patients undertaking cognitive tasks due to its simplicity, safety, and resistance to interference. When performing a cognitive task, the brain experiences an oversupply of rCBF, leading to the hemodynamic response that can be measured through fNIRS.^12^ Researchers^13^^–^^15^ found that values of oxyhemoglobin concentration in the prefrontal lobe measured by the fNIRS device were negatively correlated with HAMD scores, which were associated with depression severity, suggesting that changes in oxygen-hemoglobin are a potential biomarker for recognizing depression severity.

In recent years, several researchers^16^^,^^17^ utilized machine learning techniques to construct prediction models that differentiate between severe depression and healthy individuals based on fNIRS data, obtaining favorable classification performance. Although these studies^13^^,^^18^^–^^20^ suggest that machine learning prediction models developed based on fNIRS data serve as effective analytical tools for identifying patients with severe depression, few studies have focused on discriminating depression severity. Ramasubbu et al.^21^ developed a predictive model for distinguishing between patients with mild depression and severe depression but did not achieve high accuracy. Richter et al.^22^ proposed a diagnostic system (25 clinical anxiety or depression patients, 76 healthy control participants) and achieved an accuracy of 81.69% in distinguishing the mixed anxiety/depression group from the control group, but it only achieved a success rate of 50.66% in differentiating the anxiety group from the depression group.

Inspired by previous researches,^14^^,^^15^^,^^23^ we found that fNIRS measurement can be instrumental in distinguishing the severity of depression. In this study, we proposed a predictive model based on fNIRS data to differentiate between patients with mild and severe depression. This model provides mental health professionals with an objective and effective diagnosis method for determining the severity of depression.

## 2 Materials

### 2.1 Participants

In this study, 140 patients with drug-naïve, first-episode depression were recruited with their permission and informed consent. All subjects had no other neurological disorders (stroke, brain tumor, severe concussion, migraine, etc.), cardiovascular (myocardial infarction, arrhythmia, etc.) diseases, and no obvious impairment in vision and hearing. The experiment was conducted under the guidance of a professional psychiatrist. No data identifying the participants were recorded during the entire research process. A senior professional psychiatrist conducted interviews and observations, utilizing the HAMD^2^ scale to evaluate depressive symptom severity. According to the diagnosis of the psychiatrist, among these 140 first-episode depression patients, 58 individuals were assessed as mildly depressed, whereas the remaining 82 subjects were classified as having severe depression. More details of the participants are shown in Table 1.

**Table 1:** Subject information of our data.

	Severe depression	Mild depression
Number	82	58
Gender	54 females, 28 males	39 females, 19 males
Age (years)	32.48 ± 17.71	36.65 ± 19.97

### 2.2 Activation Task (Verbal Fluency Task)

**Fig. 1:**
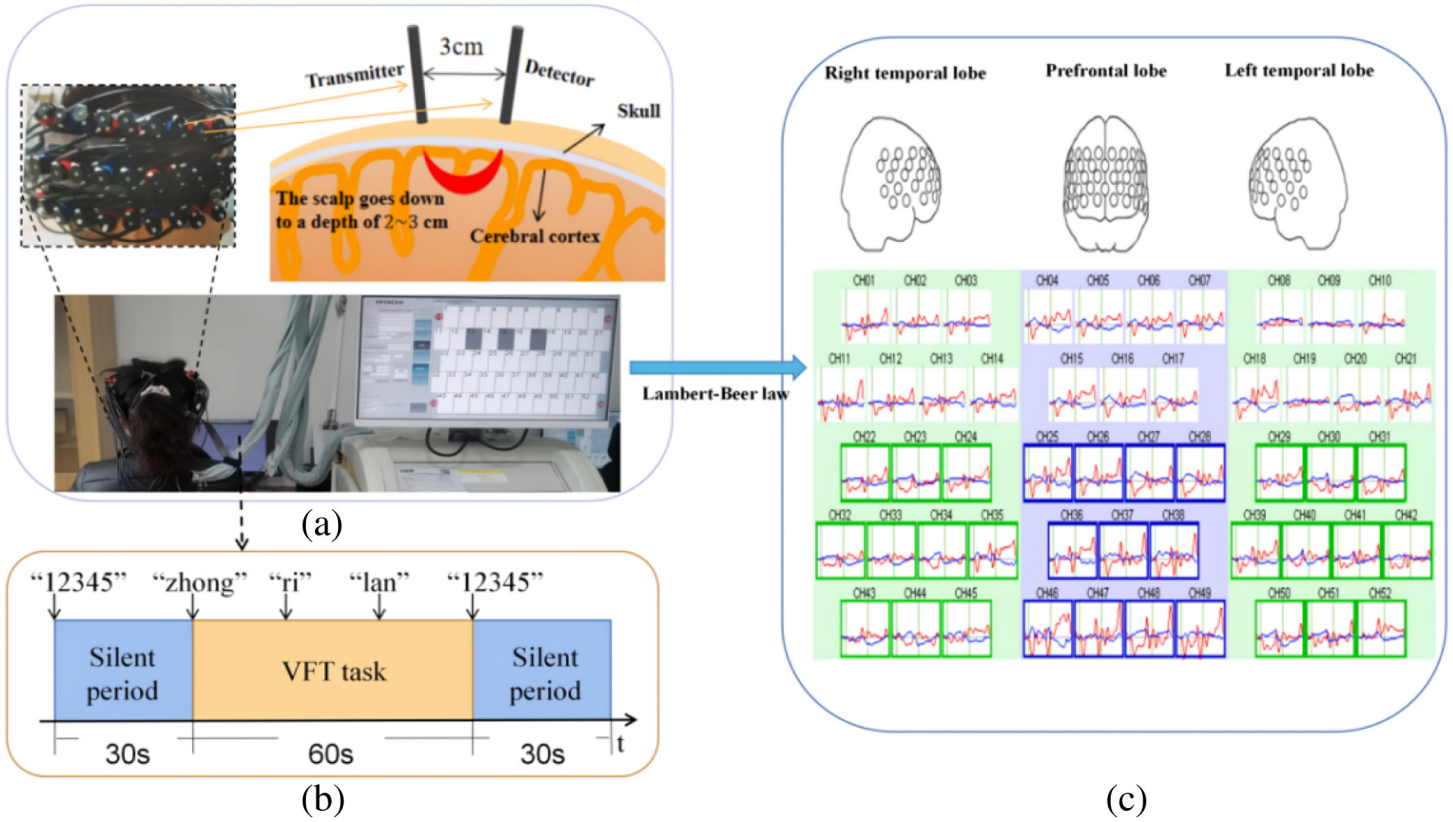
The experimental data collection process. (a) Details of the probe for the fNIRS device. (b) The VFT consisted of three stages: a 30-s pre-task baseline period, a 60-s VFT task period, and a 30-s post-task baseline period. (c) The ΔHbO and ΔHbR curves for 17 channels in the right temporal lobe, 18 channels in the prefrontal lobe, and 17 channels in the left temporal lobe.

The verbal fluency task (VFT) is a cognitive task commonly used in fNIRS research, which primarily reflects executive function and has been correlated with a number of basic neurocognitive activities (e.g., working memory, motivation, and attention).^24^ In China, the Chinese version of the VFT has been widely used in the diagnosis of psychiatric disorders as a sensitive indicator for assessing deficits in domains of cognition and executive function that depend on activation in prefrontal regions.^25^ Before the VFT task, the participants were instructed to stay in a separate, quiet room and sit on a chair, ensuring that their eyes were directly in front of the monitor at a distance of 60 cm. Participants were asked to look at the “+” symbol on the monitor and keep their heads still. At the beginning of each experiment, during a 30-s rest period before the task, a guiding voice prompt was played: “Check begins, please repeat the reading 12345.” Concurrently, the fNIRS data were recorded. Next, during a 60-s task period, the participants were required to complete three tasks involving the formation of words using the three characters “zhong,” “ri,” and “lan,” respectively. Each word formation task had a time limit of 20 s. Finally, during a 30-s resting period after the task completion, the instructional audio prompt stating, “Please repeat reading 12345 until the end of the check” was played and the fNIRS data acquisition was terminated. The entire VFT process is shown in Fig. 1(b).

### 2.3 fNIRS Data Acquisition

In the experiments, an fNIRS instrument (ETG-4100 Optical Topography System) manufactured by Hitachi Medical is utilized to obtain data. This device utilizes near-infrared light at two specific wavelengths (659±20 and 830±20  nm) to penetrate the scalp, skull, and cerebrospinal fluid, enabling irradiation of the cerebral cortex. The instrument consists of 17 transmitters and 16 detectors, forming a total of 52 channels, including 18 in the prefrontal lobes and 17 channels each in the right and left frontal lobes. The distance between each pair of transmitters and detectors was 3.0 cm, with a sampling frequency of the instrument was 10 Hz. During the VFT, neural activity leads to changes in CBF, causing changes in brain oxygen-hemoglobin (ΔHbO) and deoxygen-hemoglobin (ΔHbR).^6^ According to Lambert-Beer,^26^ changes in ΔHbO and ΔHbR can be deduced from the NIRS signals measured during the task. These changes provide a more accurate reflection of the activation state of the cerebral cortex in relation to cognitive tasks. The entire process of data collection in the experiment is shown in Fig. 1.

## 3 Methods

**Fig. 2:**
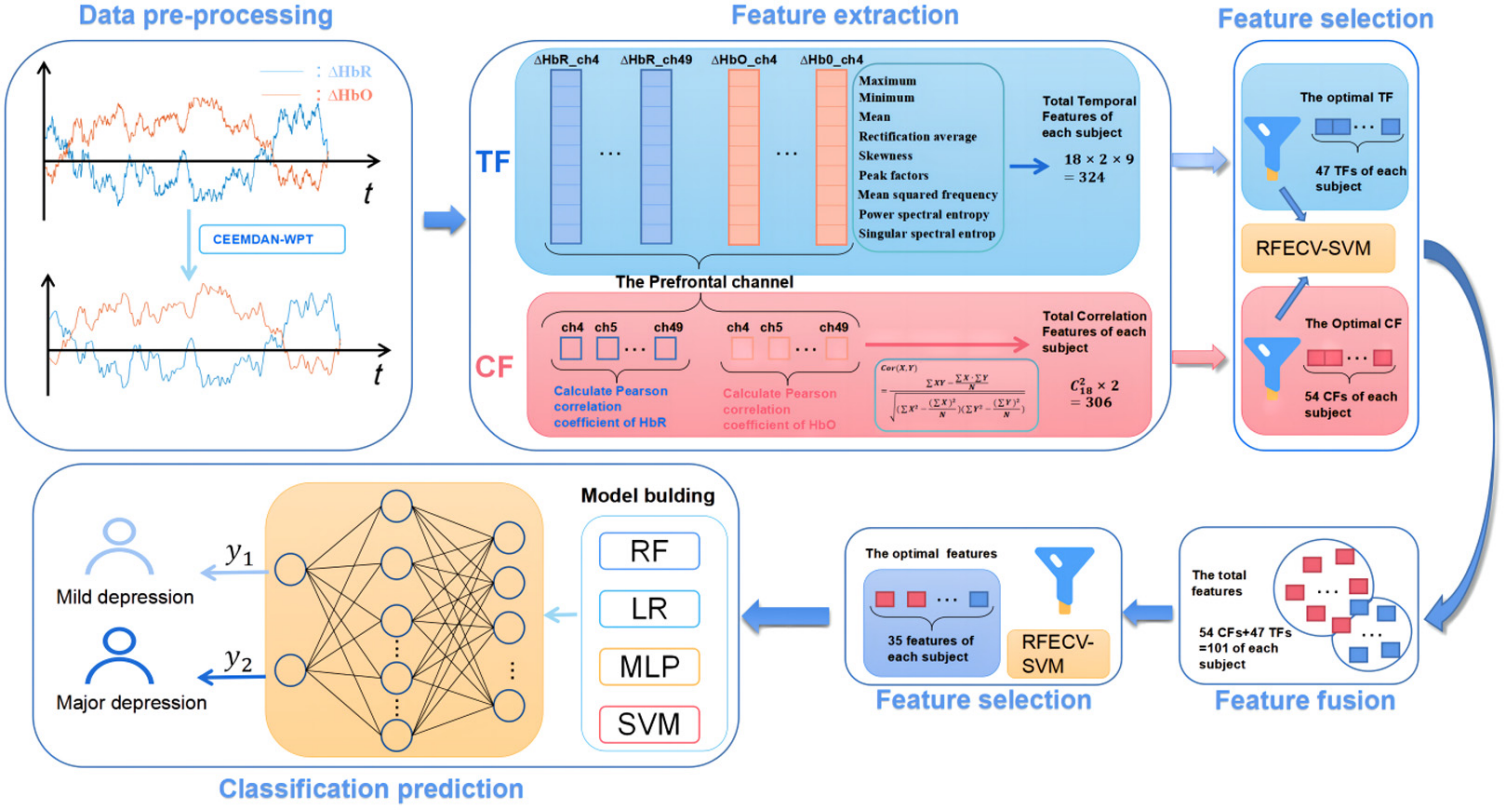
The overview of the model.

To predict the severity of depression, we developed a prediction model for mild and severe depression. The model involves data preprocessing, feature extraction, feature selection, feature fusion, and classification prediction, as shown in Fig. 2.

### 3.1 Data Pre-Processing

**Fig. 3:**
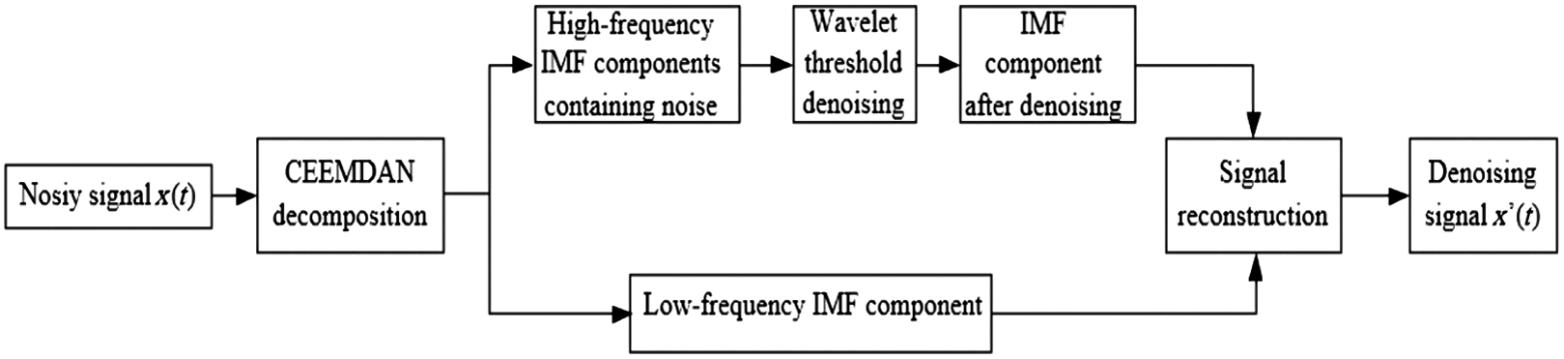
The overall process of the CEEMDAN-WPT.

We utilized the HOMER2 toolbox in MATLAB 2018b to process NIRS data and obtained the change in ΔHbO and ΔHbR using the Beer-Lambert law.^27^ These hemodynamic parameters are recognized as sensitive indicators for investigating psychiatric disorders.^28^ However, the raw hemodynamic signals often contain various types of noise that can affect the feature extraction process. Therefore, preprocessing of the fNIRS data is essential to remove noise before further analysis.^29^ We utilized a polynomial regression model to estimate linear or nonlinear trends and then subtracted this trend from the original hemoglobin concentration signal to obtain detrended data. The temporal derivative distribution repair method^30^ was employed for motion artifact correction. In addition, a third-order Chebyshev type II filter^31^ with a cutoff frequency of 0.01 Hz and a stopband frequency of 0.2 Hz was used to remove most physiological noise [e.g., respiration (0.2 to 0.4 Hz) and heartbeat (0.5 to 2.0 Hz)]. However, various artifacts, such as ambient light, electrical interference from the instrumentation, and positional movements, still contaminated the effective frequency band. In previous studies,^32^^,^^33^ empirical mode decomposition (EMD) and ensemble empirical mode decomposition (EEMD) methods have been used to eliminate motion artifacts and baseline drifts in physiological signals. In this study, to enhance the signal-to-noise ratio (SNR) and facilitate subsequent feature extraction, we applied a complete EEMD with adaptive noise-wavelet threshold (CEEMDAN-WPT) denoising method to eliminate noise contaminating the effective frequency band of fNIRS data. The flowchart of the CEEMDAN-WPT algorithm is shown in Fig. 3, and the specific implementation steps are detailed below.

1. Suppose $E_{i}(\cdot )$ represent the i’th intrinsic mode function (IMF) component operation calculated by the EMD algorithm. White noise that conforms to the standard distribution is added to the original signal, resulting in I initial signal $x^{i}(t)$:
  $$x^{i}(t)=x(t)+\varepsilon_{i}\omega^{i}(t).$$ (1)
  Here, x(t) represents the original signal, $\varepsilon_{i}$ is the white noise amplification factor, and $\omega^{i}(t)$ denotes the white noise following a standard Gaussian distribution.
2. Perform EMD decomposition on all initial signals to obtain the first modal component:
  $$\overset{¯}{{\mathrm{IMF}}_{1}(t)}=\frac{1}{I}\overset{I}{\underset{i=1}{\sum }}{\mathrm{IMF}}_{1}^{i}(t).$$ (2)
  Then, calculate the first-order remaining components:
  $$r_{1}(t)=x(t)-\overset{¯}{{\mathrm{IMF}}_{1}(t)}.$$ (3)
3. Add $E_{1}(\omega^{i}(n))$ to $r_{1}(t)$, obtaining the new signal $r_{1}(t)+\varepsilon_{1}E_{1}(\omega^{i}(n))$, and perform EMD decomposition again to calculate the second residual component:
  $$\overset{¯}{{\mathrm{IMF}}_{2}(t)}=\frac{1}{I}\overset{I}{\underset{i=1}{\sum }}E_{1}(r_{1}(t))+\varepsilon_{1}E_{1}(\omega^{i}(n)).$$ (4)
  Then, calculate the second-order residual components:
  $$r_{2}(t)=r_{1}(t)-\overset{¯}{{\mathrm{IMF}}_{2}(t)}.$$ (5)
4. Repeat the above steps until the number of poles of the obtained residual signal is less than two, indicating the end of the decomposition algorithm. At this stage, the original signal x(t) has been decomposed into
  $$x(t)=\overset{K}{\underset{k=1}{\sum }}\overset{¯}{{\mathrm{IMF}}_{k}}+R(t).$$ (6)
5. Carry out wavelet threshold denoising on the noisy high-frequency component ${\mathrm{IMF}}_{k}$:
  $$\left\{ \begin{array}{c} y=W({\mathrm{IMF}}_{k})\\ \overset{˜}{y}=\overset{˜}{D}(y,\lambda )\\ \overset{¯}{{\mathrm{IMF}}_{k}}=W(\overset{˜}{y}) \end{array}.\right.$$ (7)
  In the equation, W and $\overset{¯}{W}$ are the wavelet packet change and its inverse operation, respectively. $\tilde{D}$ and λ are the threshold function and threshold magnitude, respectively, and $\overset{¯}{{\mathrm{IMF}}_{k}}$ denotes the high-frequency component after denoising processing.
6. The denoising high-frequency component $\overset{¯}{{\mathrm{IMF}}_{k}}$ and the low-frequency component IMF are reconstructed to obtain the denoising signal $x^{\prime }(t)$.

### 3.2 Methodology for the Effective Feature and Identified Model

**Fig. 4:**
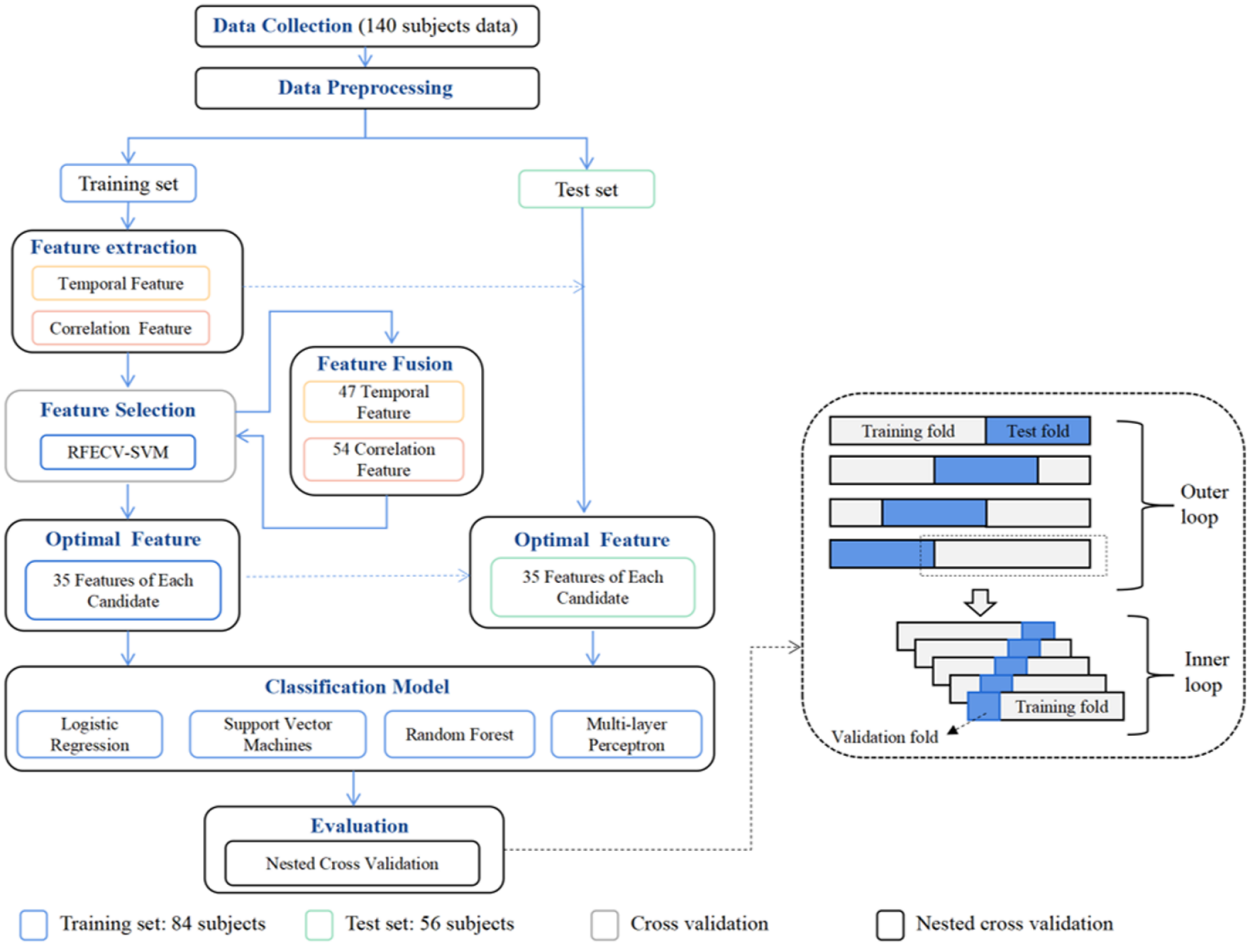
The procedure for feature selection and model training.

In our study, we employed a methodology to extract key features for effective classification of patients with mild and severe depression. This involved identifying critical features from the data obtained from channels. Subsequently, we established a recognition model using these key features to classify patients with severe depression and those with mild depression. Figure 4 shows a detailed description of feature selection and classification model construction process in this study. To ensure an objective assessment of the classification performance, the analysis was conducted exclusively on the training set within the blue-bordered box, whereas the final selected features were validated on the test set within the orange-bordered box. For comprehensive model evaluation, we utilized nested cross-validation (black-bordered box) to obtain model metrics. The principle of nested cross-validation, as shown in Fig. 4, involves two loops: an outer loop and an inner loop. The inner loop consists of cross-validation and search for the best hyperparameters of the model, such as grid search, which determines the optimal hyperparameters for the outer loop. Meanwhile, the outer loop provides training data to the inner loop while retaining some data for testing the models within the inner loop. The entire process was repeated for all possible fold combinations within the outer loop, providing a robust estimation of the model performance. This approach prevents information leakage and overfitting.

#### 3.2.1 Feature extraction

##### Temporal feature

Considering that the actual task time for VFT is only 60 s, we extracted the temporal features (TF) within this time period. These features encompass time domain characteristics, frequency domain attributes, and information entropy. The time domain features consist of maximum, minimum, mean, rectification average, skewness, and peak factors. Mean squared frequency was selected as frequency domain feature. Detailed information about the selected time and frequency domain features can be found in Table S1 in the Supplementary Material. Power spectral entropy and singular spectral entropy serve as the information entropy features, calculated as follows:

$$H_{f}=-\overset{k-1}{\underset{k=0}{\sum }}(S_{k}\overset{N}{\underset{k=1}{\sum }}S_{k})\log(S_{k}\overset{N}{\underset{k=1}{\sum }}S_{k}).$$ (8)

In the equation, $H_{f}$ represents the power spectral entropy, and $S_{k}$ denotes the energy distribution of the signal in the frequency domain:

$$H_{t}=-\sum_{i=1}^{l}(\frac{\lambda_{i}}{\sum_{i=1}^{l}\lambda_{i}})\log(\frac{\lambda_{i}}{\sum_{i=1}^{l}\lambda_{i}}),$$ (9)

where $H_{t}$ is the singular spectral entropy and $\lambda_{i}$ represents the singular value spectrum obtained through the singular value decomposition of the signal.

##### Correlation feature

In our study, the correlation among channels was extracted as the correlation features (CF). These were obtained by calculating the Pearson correlation coefficient^19^ of the change in ΔHbO and ΔHbR between 18 frontal channels of each participant. The equation for calculating the CF is as follows:

$$\mathrm{Cor}(X,Y)=\frac{\sum XY-\frac{\sum X\cdot \sum Y}{N}}{\sqrt{\left(\sum X^{2}-\frac{{\left(\sum X\right)}^{2}}{N})(\sum Y^{2}-\frac{{\left(\sum Y\right)}^{2}}{N}\right)}},$$ (10)

where X and Y represent the concentration change of hemoglobin in different channels of the participant, respectively.

Therefore, a total of 18×2×9=324 TF and $C_{18}^{2}\times 2=306$ CF can be extracted from the change curves of ΔHbO and ΔHbR in the 18 frontal channels of each participant.

#### 3.2.2 Feature selection

In the classification task, when the sample size is small and the number of features is large, it can lead to the problem of dimensionality curse and reduce the accuracy. To address this issue, recursive feature elimination with cross-validation (RFECV) method^34^ can be utilized to select the optimum features from TF and CF. The RFECV method utilizes RFE^35^ to obtain the importance of each feature and find the optimal number of features based on cross-validation accuracy. Compared to dimension reduction methods, such as PCA,^36^ the RFECV method can eliminate redundant feature information and determine the most impactful features for optimal classification performance. In our prediction model, we applied REFCV method twice. First, the optimal 47 TF and 54 CF were screened separately. Then, after fusing the optimal CF and TF selected in the first step, we further identified the best 35 features for distinguishing between mild and severe depression.

#### 3.2.3 Classification models

Given the limited number of samples in depression data, we employed machine learning techniques to construct the classification prediction. Four classical algorithms were utilized, including two supervised learning methods [logistic regression (LR)^37^ and support vector machines (SVM)^38^], an ensemble learning algorithm [random forest (RF)],^39^ and an artificial neural networks [multi-layer perceptron (MLP)].^40^ According to the participants, the dataset was split into two parts: a training set comprising 60% (49 severe depression and 35 mild depression) and a testing set comprising 40% (33 severe depression and 23 mild depression). To identify the optimal prediction model for each pattern, a grid search^41^ based on hyperparameter turning^42^ was employed to determine the model parameter with the highest accuracy.

### 3.3 Model Metrics

The denoising performance of the CEEMDAN-WPT algorithm is evaluated using two metrics: SNR and root mean square error (RMSE). SNR quantifies the relationship between signal and noise intensity, where a high SNR indicates stronger useful signals compared to noise, resulting in clearer and more reliable signals. RMSE measures the average deviation between predicted values and actual values. The equations for calculating SNR and RMSE are as follows:

$$\mathrm{SNR}=10\;\mathrm{lg}(\frac{\sum_{n=1}^{N}x^{2}(n)}{\sum_{n=1}^{N}[x(n)-y(n){]}^{2}}),$$ (11)

$$\mathrm{RMSE}=\sqrt{\frac{1}{N}\overset{N}{\underset{n=1}{\sum }}[x(n)-y(n){]}^{2}},$$ (12)

where x(n) represents the original signal, n is the the signal length, and y(n) denotes the denoised signal.

For the classification models, we calculated the area under the ROC curve (AUC) for different models to compare their performance differences. In addition, several other metrics, including specificity, sensitivity, accuracy, and F1 score, were calculated. Supplementary Material provide detailed calculation methods for these metrics.

## 4 Results and Analysis

### 4.1 Result on Denoising

**Fig. 5:**
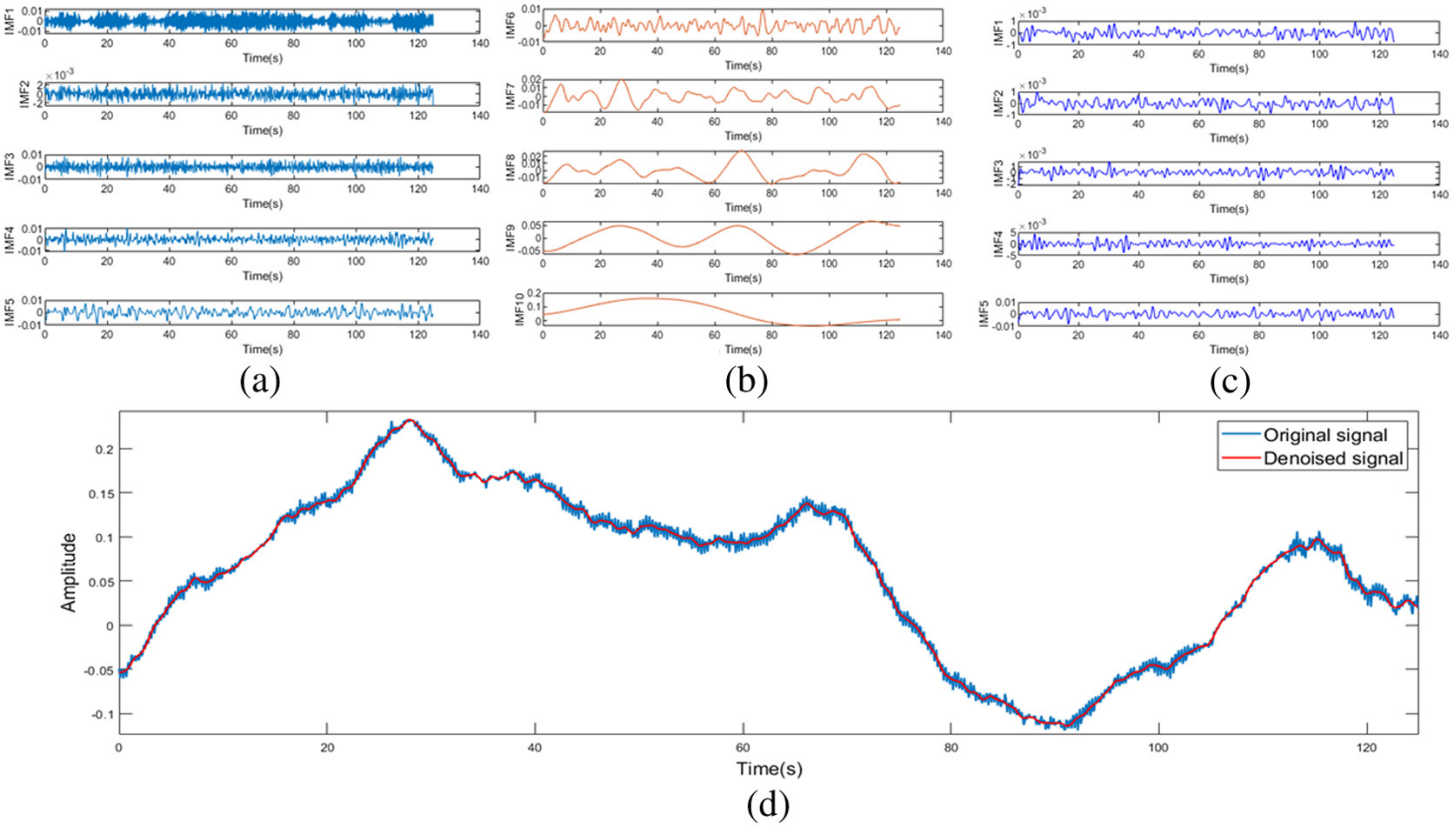
Noise reduction signal comparison. (a) IMF1 through IMF5 represent the high-frequency components, (b) IMF6 through IMF10 represent the low-frequency components; (c) the high-frequency components after denoising. (d) Comparison diagram of the original signal and the denoised signal.

**Fig. 6:**
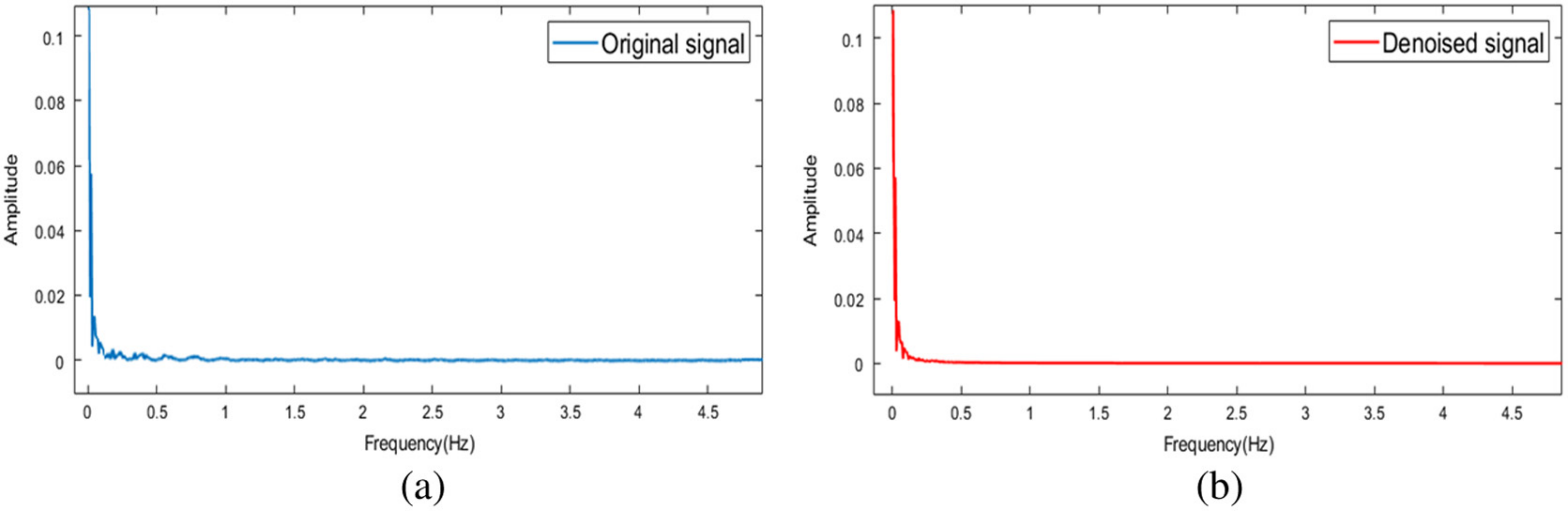
The spectrum of the original signal and the denoised signal; (a) represents the one-sided spectrum of the original signal and (b) illustrates the one-sided spectrum of the denoised signal.

As shown in Fig. 5, the original signal is decomposed into five high-frequency components and five low-frequency components. The high-frequency components denoised by wavelet packet threshold and low-frequency components are reconstructed into a new signal, which is visibly smoother. Figure 6 shown the spectrum of the original signal and the signal processed using the CEEMDAN-WPT method. In Fig. 6(a), it can be observed that even after filtering, a small amount of noise remains mixed within the effective frequency range. However, our proposed method effectively removes this noise while retaining the information relevant to neural activity. In Table 2, compared to EMD,^43^ the CEEMDAN-WPT method significantly improves SNR and reduces the RMSE. Compared to wavelet filtering,^44^ although there is a slight difference in RMSE, the SNR is improved by 4.1 dB.

**Table 2:** Performance comparison of three algorithms.

Method	SNR	RMSE
EMD	5.8842	0.1675
Wavelet filtering	16.8702	0.0297
CEEMDAN-WPT	20.9767	0.0258

### 4.2 Result on Feature Selection

**Fig. 7:**
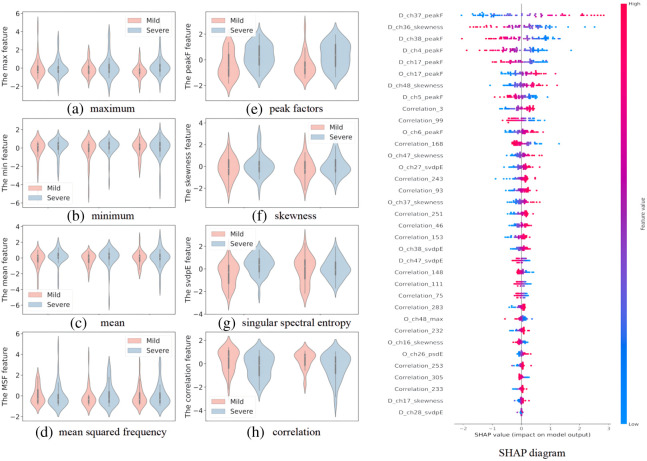
Violin and SHAP diagram about features. (a) through (d) represent the maximum, minimum, average, and root mean square frequency, respectively. (e) through (h) correspond to the peak factor, skewness, singular spectrum entropy, and correlation coefficient, respectively. (a) through (d) visualize the features that were excluded, while (e) through (h) showcase features selected by RFECV. The SHAP diagram ranks the importance of 35 optimal features, depicting the distribution of their impact on the prediction results along the X-axis. Each point represents a sample, and the color represents the feature value. For instance, the first row indicates that high peak factor has a positive impact on the prediction results, whereas low peak factor has a negative impact.

Through the RFECV algorithm, we identified 35 optimal features that are related to the severity of depression. As shown in Fig. 7, we plotted some violin figures regarding the features. It can be seen from the figure that there are significant differences between mild depression and severe depression subjects in terms of peak factor, skewness, singular spectral entropy, and correlation in certain channels. Conversely, the differences in maximum, minimum, mean, and mean square frequency are relatively small. The SHAP diagram in Fig. 7 demonstrated that peak factor and skewness in certain channels have a significant impact on the prediction results, whereas the influence of maximum, minimum, average, and mean square frequency is relatively minor.

### 4.3 Activation Analysis

**Fig. 8:**
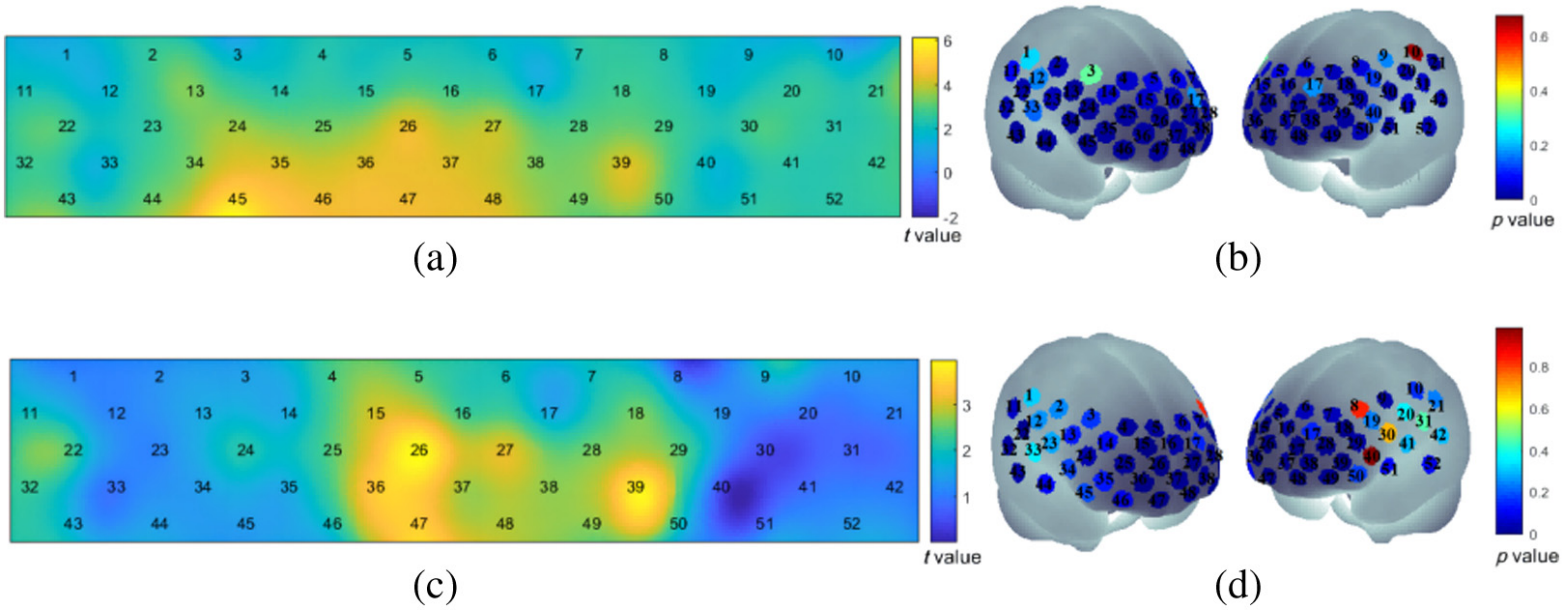
Analysis of channel activation status and activation intensity in mild and severe depression. (a) The heat map of t-values representing the brain channel activations during the VFT, where the blue regions indicate non-activated channels. (b) Dark blue channels (p<0.05) indicate activated channels. (c) The heat map of t-values compares the intensity of activation between the mild depression and severe depression groups, with blue regions indicating no significant differences between the two groups. (d) Dark blue channels (p<0.05) represent significant differences between the mild depression and severe depression groups.

Individual-level statistical analysis was performed on preprocessed fNIRS data using the general linear model to calculate beta values representing brain activation intensity during the VFT task. The activation levels of individual channels were evaluated using a one-sample t-test, and the heat map of channel activation t-values is presented in Fig. 8(a). Channels, such as ch1, ch3, ch9, ch10, ch11, ch12, ch17, ch19, ch33, and ch40, did not show significant activation during the VFT task, whereas the remaining channels exhibited significant activation. In Fig. 8(a), 52 channels were mapped onto the brain, with non-activated channels mainly located in the left and right temporal lobes. To compare brain activation intensity between the severe depression and mild depression groups, a paired t-test was conducted. The results indicated that, during the VFT task, the activation levels in the mild depression group were higher than those in the severe depression group. Notably, there was a significant difference in channel activation intensity in the frontal lobe region, as depicted in Figs. 8(c) and 8(d). The Supplementary Material contain detailed results of both the one-sample and paired sample t-tests.

### 4.4 Result on Classification Model

We evaluated the TF, the CF, and the fusion of both as inputs for the four classification models and validated the results on the test set, as shown in Table 3. Across all models, the fusion of temporal and CFs consistently outperformed individual temporal or CFs. Furthermore, in LR, MLP, and SVM models, the AUC of the fusion features was greater than 0.9, indicating its ability to better distinguish between mild and severe depression. Notably, the fusion features of temporal and correlation fusion features showed outstanding performance in the SVM classification model, with an accuracy of 92.8%, a sensitivity of 91.6%, a specificity of 93.7%, and an F1-score of 92.7%.

**Table 3:** The results of classification models.

Method		Classification metrics				
Model	Feature	AUC	Accuracy	Sensitivity	Specificity	F1-score
RF	TF	0.757	0.678	0.333	0.937	0.619
	CF	0.645	0.642	0.333	0.875	0.590
	TCF	0.739	0.714	0.50	0.875	0.688
LR	TF	0.770	0.714	0.50	0.875	0.688
	CF	0.911	0.821	0.833	0.812	0.819
	TCF	0.927	0.857	0.666	1.0	0.844
MLP	TF	0.916	0.821	0.583	1.0	0.801
	CF	0.906	0.821	0.750	0.875	0.815
	TCF	0.968	0.892	0.750	1.0	0.885
SVM	TF	0.942	0.857	0.750	0.937	0.850
	CF	0.895	0.821	0.750	0.875	0.815
	TCF	0.963	0.928	0.916	0.937	0.927

Abbreviation: RF, random forest; LR, logistic regression; MLP, multi-layer perceptron; SVM, support vector machine; TF, temporal features; CF, correlation features; TCF, temporal and correlation fusion features.

## 5 Discussion

Cerebral blood flow (CBF) is associated with brain activity and metabolism. During cognitive tasks, rCBF increases^12^ while significant changes in CBF occur in patients with depression, particularly in the prefrontal region. Specifically, rCBF is lower in patients with mild depression compared to the normal control group, whereas it is higher in patients with moderate to severe depression compared to the rCBF in most cortical regions of the normal control group.^45^^,^^46^ The changes in rCBF can lead to hemodynamic responses, which can be measured using an fNIRS device, making it an important tool for studying depression. However, the fNIRS device is sensitive to motion artifacts and noise, posing a challenge in filter out noise while retaining depression-related information. Some researchers^47^ attempted to use wavelet filtering to remove the motion artifacts from fNIRS data and compared it with other methods for correcting motion artifacts, such as principal component analysis, spline interpolation, and Kalman filtering. The results indicated that wavelet filtering had better performance. However, in this paper, the CEEMADN-WPT method was utilized to remove noise from fNIRS data, which is a more powerful technique. Compared to signals filtered by wavelet filtering, the signal filtered by CEEMADN-WPT had higher SNR (as shown in Table 2). With this denoising method, we effectively reduced the noise mixed in the valid frequency band in fNIRS data, thus preserving information related to depression. Shallow tissues, such as the scalp, skull, and meninges, are abundant in capillaries. The concentration of hemoglobin within these capillaries fluctuates due to respiration, heartbeat, and task-related autonomous neural activity. When near-infrared light penetrates these shallow tissues, changes in hemoglobin concentration lead to variations in fNIRS light attenuation, referred to as shallow physiological noise. The “short-separation” method (channels where the emitter-detector distance is below 1.0 cm) is recognized as an effective approach of removing shallow noise. This method involves using additional short-separation fNIRS channels to record shallow physiological noise and then subtract it from the signal. The absence of physiological noise data during our data collection process has posed a certain limitation on our preprocessing of fNIRS data. In future studies, we plan to collect both fNIRS data and physiological noise data to better capture data related to brain neural activity.

In this study, temporal and channel CFs extracted from fNIRS data of the prefrontal lobe of patients with severe and mild depression were compared. Our findings suggest that using TFs as inputs leads to higher accuracy compared to using only CFs. Furthermore, the fusion of temporal and CFs obtained the highest accuracy with an AUC of 96.3% on SVM models. These results indicate that studying the functional connectivity of the brain alone is insufficient in distinguishing between patients with mild and severe depression. By combining the temporal and CFs of fNIRS data, the classification accuracy can be improved. Through feature visualization, significant differences can be observed between patients with mild depression and those with severe depression in certain TFs, such as peak factor, skewness, and singular spectral entropy. In addition, the peak factor and skewness of ΔHbO and ΔHbR curves play a crucial role in the output of the model. The research results indicate that temporal and channel CFs extracted from ΔHbO and ΔHbR are important for distinguishing the severity of depression, and these features have the potential to serve as biomarkers for distinguishing between mild and severe depression, which is consistent with previous research results.^13^

The findings of our research have several potential applications in clinical services. The diagnostic support system based on fNIRS that we propose provides a non-invasive, objective, and quantitative method for assessing the severity of depression, which can serve as a supplement to traditional diagnostic methods, such as clinical interviews or self-report questionnaires. By combining fNIRS technology and machine learning algorithms, our model can extract valuable information from brain activity patterns that are difficult to detect using traditional methods. These results can serve as an auxiliary tool for psychiatrists, helping them accurately assess the severity of depression in patients and develop more personalized treatment plans. In addition, our research outcomes can be utilized for monitoring treatment progress. By regularly conducting fNIRS tests, clinicians can objectively understand changes in patient’s conditions and make timely adjustments to treatment plans for better therapeutic efficacy.

It is important to note that this study has several limitations. First, the small sample size of 140 participants, including 82 cases of severe depression and 58 cases of mild depression, may have limited the precision of our model. A larger dataset is needed to improve the effectiveness of the model. The participants in the study were drug-naive, first-episode depression patients receiving outpatient treatment at the Mental Health Center in Chongqing City. Therefore, our findings are not influenced by medication, or previous treatments, as these data were not included in the analyzed dataset. However, our study did not consider potential differences among the participants, such as long-term smoking, alcohol consumption, the number of past depressive episodes, or a family history of mental illness, all of which could have had an impact on the severity of depression. In addition, the diagnostic data were obtained from a single psychiatric research center, and the generalizability of our model to other hospitals has not been validated. Furthermore, due to the small sample size, patients with moderate depression were not included in the study, necessitating further research in this area. Future work will involve collecting a larger sample size to investigate the severity of depression and analyzing clinical information among participants. We also plan to develop a deep learning-based predictive model for classifying mild, moderate, and severe depression and embed the model into the software used by hospitals. By connecting to the fNIRS database and integrating clinical information from patients, we aim to obtain more accurate and personalized diagnostic results through big data analysis, providing psychiatrics with a more reliable and efficient tool to support their decision-making in depression treatment.

## 6 Conclusions

This paper proposes a depression severity prediction model based on the temporal and CFs extracted from fNIRS data, achieving high classification accuracy for mild and severe depression. As an objective auxiliary tool, this model can improve the diagnostic efficiency of depression severity and assist doctors in clinical diagnosis, which is of great significance for the treatment of depressed patients.

## Supplementary Material



## Data Availability

The data and materials that support the findings of this study are available upon request. Due to privacy and confidentiality concerns, the raw data cannot be publicly shared. However, interested researchers can contact Zhiyong Huang at zyhuang@cqu.edu.cn to request access to the data used in this study. The code used to generate the results and figures is available in a Github repository: https://github.com/liumanliu/fNIRS_depression.
