# Supplementary material for: Functional near-infrared spectroscopy-based diagnosis support system for distinguishing between mild and severe depression using machine learning approaches
Source: Neurophotonics. 2024 Apr 24;11(2):025001. doi: 10.1117/1.NPh.11.2.025001 (PMC11042567; doi:10.1117/1.NPh.11.2.025001)
Supplement: Supplementary file 1 [file NPh_011_025001_SD001.pdf]

Supplementary Materials

1. Experiment between Healthy Controls and Participants with Mild Depression

1.1 Objective

The objective of this experiment was to assess the performance of the model presented in the main text in differentiating mild depression from healthy controls using functional near-infrared spectroscopy (fNIRS) data.

1.2 Participants

Due to the limited availability of healthy volunteers, our control group comprised only 16 individuals. All participants provided appropriate consent and were fully informed about the study. None of the participants had any other neurological conditions (such as stroke, brain tumors, severe concussion, or migraines) or cardiovascular diseases (such as myocardial infarction or arrhythmias). Additionally, their auditory and visual functions were normal. Their healthy condition were evaluated using the Hamilton Depression Rating Scale (HAMD) and assessed by psychiatric professionals. To mitigate the impact of a small sample size, we randomly selected subsets of fNIRS data from both mild depression patients and healthy controls for the model training process. As a result, our dataset consisted of 56 participants (40 with mild depression and 16 healthy controls). More details of the participants are shown in Table 1.

Table 1: Subject information of our data

	**Mild depression**	**Healthy controls**
Number	40	16
Gender	29 females, 11 males	11 females, 5 males
Age(years)	43.37 ±21.42	32.55±17.95

1.3 Data Collection and Pre-processing

The fNIRS data were collected from participants during the verbal fluency task (VFT). The data collection and pre-processing procedure were consistent with those described in the main study.

1.4 Feature Extraction

In the main study, our model was employed to extract a set of 35 optimal features for further analysis. These features were carefully selected based on their relevance and significance in differentiating between mild and severe depression. This set of features was also utilized in comparative experiments between individuals with mild depression and a control group of healthy individuals. The selection of these features was grounded in their ability to effectively distinguish varying degrees of depression severity. Their inclusion in the comparative experiments allowed us to assess the differences in brain activity patterns between individuals with mild depression and those without any

depressive symptoms.

1.5 Model Evaluation

To evaluate the performance of our model in classifying depressive status, we combined the fNIRS data from both groups (mild depression and healthy controls). The dataset was randomly divided into training and testing sets, with a ratio of 6:4. This ensured a robust evaluation of the model's effectiveness in accurately classifying the depressive status of the participants. Specifically, we utilized the training set to train the model on the combined fNIRS data, allowing it to learn the patterns and characteristics associated with different depressive statuses. Once trained, the model was then tested on the independent testing set to assess its classification performance. The metrics used to assess the performance of this model are the same as the model metrics in the previous main text, including AUC, specificity, sensitivity, accuracy, and F1 score. By comparing these metrics, we can measure the effectiveness of our model in accurately identifying the severity of depression.

1.6 Results

We evaluated the time features, correlation features, and the fusion of both as inputs for four classification models, and validated the results on the test set, as shown in Table 2. The fusion feature, when used as input, demonstrated excellent performance on both SVM and MLP algorithms. This indicates that our model can effectively distinguish between the healthy control group and individuals with mild depression, further validating its effectiveness in assessing the severity of depression.

Table 2: The results of classification models

Method		classification metrics				
**Model**	**Feature**	**AUC**	**Accuracy**	**Sensitivity**	**Specificity**	**F1-Score**
**RF**	**TF**	0.754	0.695	0.0	**1.0**	0.410
	**CF**	0.741	0.782	0.285	**1.0**	0.654
	**TCF**	0.776	0.695	0.0	**1.0**	0.410
**LR**	**TF**	0.606	0.652	0.142	0.875	0.488
	**CF**	0.937	0.826	0.428	**1.0**	0.744
	**TCF**	0.821	0.782	0.714	0.812	0.752
**MLP**	**TF**	0.919	0.913	1.0	0.875	0.904
	**CF**	0.946	0.826	0.428	**1.0**	0.744
	**TCF**	**0.973**	**0.956**	**0.857**	**1.0**	**0.946**
**SVM**	**TF**	0.678	0.652	0.428	0.75	0.589
	**CF**	0.937	0.826	0.428	**1.0**	0.744
	**TCF**	**0.973**	**0.956**	**0.857**	**1.0**	**0.946**

Abbreviation* RF: Random Forest; LR: Logistic Regression; MLP: Multi-layer perceptron; SVM: Support Vector Machine; TF: Temporal Features; CF: Correlation Features; TCF: Temporal and Correlation Fusion features.

1.7 Discussion

Our model is primarily designed to differentiate the severity of depression, demonstrating high accuracy in distinguishing between severe and mild depression. In this supplementary experiment, despite the limited number of participants in the healthy control group, preliminary results indicate the model's ability to effectively differentiate mild depression from healthy individuals using fNIRS data. However, it is important to note that further research with a larger sample size is essential to validate these initial findings and enhance the reliability of the model. To comprehensively evaluate our model, future studies should consider expanding the sample size to include more healthy participants to ensure robust and consistent results. Additionally, we need to consider other clinical factors that may influence the severity of depression to better interpret our predictive outcomes.

2. The temporal features

The temporal features used in our study include Maximum, Minimum, Mean, Rectification Average, Skewness, Peak Factors, Mean Squared Frequency, Power Spectral Entropy, and Singular Spectral. These features provide important insights into the temporal characteristics of the data.

Table 3 The explanations and formulas of temporal features

Feature	Description	Mathematical formulas
Maximum	Maximum refers to the highest value obtained from the data collected by each channel during the 60-second VFT experiment.	$X_{\max} = \max\{x_i\}$
Minimum	Minimum refers to the lowest value of data collected by each channel during the 60-second recording period.	$X_{min} = min\{x_i\}$
Mean	Mean refers to the average of the data collected from each channel during the 60-second fNIRS data recording.	$\bar{x} = \frac{1}{N}\sum_{i=1}^{N} x_i$
Rectification average	Rectification average refers to calculating the average value of all points after taking their absolute values.	$\bar{X} = \frac{1}{N}\sum_{i=1}^{N} \lvert x_i \rvert$
Skewness	Skewness is a numerical feature used to measure the degree of asymmetry in a given data distribution, which reflects the direction and degree of skewness in the data distribution.	$\alpha = \frac{1}{N}\sum_{i=1}^{N} x_i^3$

Peak factors	The peak factor is the ratio of the peak to the root mean square of a given data, used to measure the extreme degree of the peak value of the data.	$C = \frac{\max\{\lvert x_i \rvert\}}{\sqrt{\frac{1}{N}\sum_{i=1}^{N} x_{\mathrm{i}}^2}}$
Mean squared frequency	The mean square frequency refers to the mean square value of the power spectral density. It is used to measure the energy distribution of signals at different frequencies and reflects the characteristics of signals in the frequency domain.	$S_3 = \frac{\sum_{k=1}^{k} f_k^2 s(k)}{\sum_{k=1}^{k} s(k)}$
Power spectral entropy	Power spectral entropy is a statistical measure used to measure the spectral distribution of a signal or data. It is based on the power spectral density function of the signal, and evaluates the complexity and information content of the signal by calculating the probability density distribution of the spectrum.	$H_f = -\sum_{k=0}^{k-1}(S_k \sum_{k=1}^{N} S_k) \log\left(S_k \sum_{k=1}^{N} S_k\right)$
Singular spectral entropy	Singular spectral entropy describes the complexity and irregularity of time series by calculating the contribution entropy of singular components. It can segment time series to identify different patterns and trends in the time series, which is helpful for data analysis and prediction.	$H_t = -\sum_{i=1}^{l}\left(\frac{\lambda_i}{\sum_{i=1}^{l}\lambda_i}\right)\log\left(\frac{\lambda_i}{\sum_{i=1}^{l}\lambda_i}\right)$

In the formula, H_f represents the power spectral entropy, and S_k represents the energy division of the signal in the frequency domain. H_t is the singular spectral entropy and λ_i is the singular value spectrum obtained by the singular value decomposition of the signal.

3. Model metrics

Based on the confusion matrix in Table 4 below, the model metrics (model metrics)

are analyzed and presented in Table 5.

Table 4: Confusion matrix

	Actual Positive	Actual Negative
Predicted Positive	True Positive (TP)	False Positive (FP)
Predicted Negative	False Negative (FN)	True Negative (TN)

Table 5: Classification model metrics

**Metrics**	**Description**	**Mathematical formulas**
Signal-to-noise ratio (SNR)	SNR is an indicator that measures the relationship between signal and noise intensity. High SNR means useful signals are stronger than noise, representing clearer and more reliable signals.	$10\,lg(\frac{\sum_{n=1}^{N} x^2\,(n)}{\sum_{n=1}^{N}[x(n)-y(n)]^2})$
Root mean squared error (RMSE)	RMSE represents the average deviation between the predicted value and the actual value.	$\sqrt{\frac{1}{N}\sum_{n=1}^{N}[x(n)-y(n)]^2}$
Accuracy	Both positive and negative samples are classified accurately.	$\frac{TP+TN}{TP+FP+FN+TN}$
Sensitivity	The proportion of correctly classified positive samples to the total positive samples.	$\frac{TP}{TP+FN}$
Specificity	The proportion of correctly classified negative samples to the total negative samples.	$\frac{TN}{FP+TN}$
F1-Score	The harmonic average of precision and recall.	$\frac{2TP}{2TP+FP+FN}$
AUC	It is the area under the receiver operating characteristic curve (ROC), indicating the comprehensive performance of the classifier.	

4. Results of T-test

Table 6: Results of one-sample t-test

**The left temporal lobe**			**The prefrontal lobe**			**The Right temporal lobe**		
channel	t	p	channel	t	p	channel	t	p
**Ch1**	**1.253**	**0.221**	Ch4	2.308	0.028	Ch8	2.221	0.035
Ch2	2.443	0.021	Ch5	2.096	0.046	**Ch9**	**1.461**	**0.158**
**Ch3**	**1.012**	**0.32**	Ch6	2.073	0.048	**Ch10**	**0.418**	**0.678**
**Ch11**	**1.945**	**0.064**	Ch7	2.567	0.016	Ch18	2.722	0.011
**Ch12**	**1.441**	**0.160**	Ch15	2.288	0.032	**Ch19**	**1.691**	**0.112**
Ch13	3.404	0.002	Ch16	2.573	0.015	Ch20	2.540	0.018
Ch14	2.157	0.039	**Ch17**	**1.429**	**0.166**	Ch21	3.43	0.002
Ch22	3.052	0.005	Ch25	3.298	0.003	Ch29	2.719	0.011
Ch23	2.421	0.022	Ch26	4.451	<0.001	Ch30	2.925	0.007
Ch24	3.686	<0.001	Ch27	3.973	<0.001	Ch31	2.336	0.027
Ch32	2.487	0.019	Ch28	3.027	0.005	Ch39	4.329	0.001
**Ch33**	**1.503**	**0.143**	Ch36	4.438	0.001	**Ch40**	**1.609**	**0.118**
Ch34	3.342	0.002	Ch37	4.435	0.001	Ch41	2.311	0.029
Ch35	4.437	0.001	Ch38	3.395	0.002	Ch42	2.864	0.007
Ch43	3.584	0.001	Ch46	4.627	<0.001	Ch50	2.443	0.021
Ch44	3.34	0.002	Ch47	4.591	<0.001	Ch51	2.617	0.014
Ch45	6.16	<0.001	Ch48	4.392	0.001	Ch52	2.44	0.022
			Ch49	3.123	0.004			

Bold indicates(p<0.05) channels that are not activated.

Table 7: Results of paired t-test

**The left temporal lobe**			**The prefrontal lobe**			**The Right temporal lobe**		
channel	t	p	channel	t	p	channel	t	p
**Ch1**	**1.003**	**0.319**	Ch4	2.379	0.020	**Ch8**	**0.2**	**0.84**
**Ch2**	**1.149**	**0.254**	Ch5	2.299	0.024	Ch9	2.261	0.026
**Ch3**	**1.381**	**0.171**	Ch6	1.997	0.049	**Ch10**	**1.423**	**0.159**
**Ch11**	**1.892**	**0.062**	Ch7	2.02	0.047	Ch18	2.503	0.014
**Ch12**	**1.127**	**0.263**	Ch15	2.954	0.004	**Ch19**	**1.187**	**0.239**
**Ch13**	**1.507**	**0.136**	Ch16	2.218	0.029	**Ch20**	**0.924**	**0.358**
**Ch14**	**1.533**	**0.129**	**Ch17**	**1.457**	**0.149**	**Ch21**	**1.187**	**0.239**
Ch22	2.622	0.010	Ch25	2.425	0.018	Ch29	2.26	0.027
**Ch23**	**1.093**	**0.278**	Ch26	3.974	<0.001	**Ch30**	**0.403**	**0.687**
Ch24	2.115	0.038	Ch27	3.115	0.003	**Ch31**	**0.765**	**0.446**
Ch32	2.241	0.028	Ch28	2.649	0.01	Ch39	3.893	<0.001
**Ch33**	**0.951**	**0.345**	Ch36	3.435	0.001	**Ch40**	**0.01**	**0.992**
**Ch34**	**1.349**	**0.181**	Ch37	2.758	0.007	**Ch41**	**1.041**	**0.302**
**Ch35**	**1.493**	**0.139**	Ch38	2.713	0.008	**Ch42**	**1.039**	**0.303**
Ch43	2.077	0.042	**Ch46**	**1.655**	**0.103**	**Ch50**	**1.362**	**0.178**
**Ch44**	**1.574**	**0.12**	Ch47	3.641	<0.001	**Ch51**	**1.556**	**0.125**
**Ch45**	**1.332**	**0.187**	Ch48	2.888	0.005	**Ch52**	**1.613**	**0.111**
			Ch49	2.509	0.015			

Bold indicates($p<0.05$) channels where there is no significant difference in activation strength.
